# Regulatory functions and pathological relevance of the *MECP2* 3′UTR in the central nervous system

**DOI:** 10.1186/s13619-015-0023-x

**Published:** 2015-10-28

**Authors:** Heather McGowan, Zhiping P. Pang

**Affiliations:** Department of Neuroscience and Cell Biology, Child Health Institute of New Jersey, Rutgers University Robert Wood Johnson Medical School, 89 French Street, Room 3277, New Brunswick, NJ 08901 USA

**Keywords:** Methyl-CpG-binding protein 2, 3' untranslated region, Autism, Rett syndrome

## Abstract

Methyl-CpG-binding protein 2 (MeCP2), encoded by the gene *MECP2*, is a transcriptional regulator and chromatin-remodeling protein, which is ubiquitously expressed and plays an essential role in the development and maintenance of the central nervous system (CNS). Highly enriched in post-migratory neurons, MeCP2 is needed for neuronal maturation, including dendritic arborization and the development of synapses. Loss-of-function mutations in *MECP2* cause Rett syndrome (RTT), a debilitating neurodevelopmental disorder characterized by a phase of normal development, followed by the progressive loss of milestones and cognitive disability. While a great deal has been discovered about the structure, function, and regulation of MeCP2 in the time since its discovery as the genetic cause of RTT, including its involvement in a number of RTT-related syndromes that have come to be known as MeCP2-spectrum disorders, much about this multifunctional protein remains enigmatic. One unequivocal fact that has become apparent is the importance of maintaining MeCP2 protein levels within a narrow range, the limits of which may depend upon the cell type and developmental time point. As such, MeCP2 is amenable to complex, multifactorial regulation. Here, we summarize the role of the *MECP2* 3' untranslated region (UTR) in the regulation of MeCP2 protein levels and how mutations in this region contribute to autism and other non-RTT neuropsychiatric disorders.

## Introduction

In 1999, Huda Zoghbi and her colleagues discovered that *MECP2*, which codes for methyl-CpG-binding protein 2 (MeCP2), is the gene that is mutated in Rett syndrome (RTT) [1]. It is now known from a decade and a half of study that MeCP2 is a multifunctional protein that plays a complex, yet essential role, in the development and maintenance of the central nervous system (CNS). The diversity of MeCP2 function includes, but may not be limited to: transcription regulation [2–4], chromatin-remodeling and histone modification [5–7], and regulation of messenger RNA (mRNA)-splicing [8, 9] and microRNA (miRNA)-processing [10]. These molecular functions manifest themselves on a cellular level in ways that are not completely understood but ultimately result in proper neural cell differentiation [11], neuronal maturation [12–14], dendritic arborization and spine formation [15–17], and adequate production of synaptic proteins and receptors [18, 19]. Moreover, MeCP2 exerts both cell autonomous and non-cell autonomous effects on neurons [20]. Once thought to be exclusive to neurons in the CNS, we now also know that glial cells, including astrocytes and oligodendrocytes, express MeCP2 and require it to adequately support the morphological and functional development of neurons. In turn, glial cells have been implicated as key players in the pathophysiology of RTT and MeCP2-related disorders [21–25].

In most cases, loss of MeCP2 function in females results in classic RTT [26]. RTT is an X-linked neurodevelopmental disorder that is characterized by 6–18 months of normal development, followed by a stagnation and eventual regression of developmental milestones. Affected individuals exhibit a myriad of characteristic signs and debilitating symptoms, including microcephaly, intellectual disability, autistic features, overall growth retardation and weight loss, hypotonia, loss of motor coordination, autonomic dysfunction, breathing irregularities and apneas, and replacement of purposeful hand movements with stereotypies such as wringing, clapping, or flapping [27, 28]. In males, RTT-causing mutations most often result in severe neonatal encephalopathy [29, 30]; however, in rare cases, these same mutations can cause classic RTT in males with Klinefelter syndrome (47, XXY) or somatic mosaicism [31, 32]. Loss-of-function mutations that do not cause RTT produce a host of neuropsychiatric abnormalities in both males (e.g., mental retardation, bipolar disorder, schizophrenia, PPM-X syndrome) [30, 33–36] and females (e.g., atypical RTT, mental retardation, Angelman-like syndrome, autism) [37–41]. In addition, increases in MeCP2 dosage also lead to profound dysfunction. Duplications of the gene locus results in MeCP2 duplication syndrome, which is a progressive neurodevelopmental disorder with RTT-like features that occurs most often in males [42, 43].

While it is intriguing that dramatic losses and gains in the *MECP2* gene dosage both result in a similar, debilitating phenotype, it has also been demonstrated that a hypomorphic *MECP2* allele that expresses 50 % of the wild type gene level also produces an RTT-like syndrome in mice [44], thus demonstrating a need for MeCP2 protein levels to be maintained within a narrow margin to ensure proper neurological development. Accordingly, there is also evidence that MeCP2 levels are reduced in other neurodevelopmental disorders, including autism, trisomy 21, fragile X syndrome, Angelman syndrome, and Prader-Willi syndrome [37, 45]. Therefore, it is not surprising that *MECP2* expression is subject to intense, complex regulation at virtually every level from DNA to protein. While the overall expression patterns for MeCP2 in the developing nervous system has been elucidated, there is not always a clear correlation between mRNA and protein expression, and the various and complex mechanisms governing the transcript-protein balance are not completely understood [12, 13, 37, 46–50]. The clinical relevance for elucidating these processes is particularly crucial, not only for providing a more complete understanding of the heterogeneity in the clinical presentation and treatment potential of RTT and related MeCP2-spectrum disorders, but also for those above-mentioned developmental disorders in which MeCP2 expression is dysregulated in the absence of an obvious mutation. In this review, we will summarize the current understanding of the spatio-temporal expression patterns of MeCP2 throughout brain development, as well as the regulatory mechanisms that control the specificity of these patterns, focusing on the regulatory potential of the highly conserved 3′ untranslated region (3′UTR). We will seek to establish the 3′UTR as an important potential contributor in establishing and maintaining homeostatic levels of MeCP2 expression in a developmentally appropriate manner. Finally, we will discuss the clinical relevance of these regulatory mechanisms as they are currently understood and their implications for potential therapeutic strategies.

### *MECP2* expression

During development, MeCP2 protein is expressed at low levels throughout the brain prenatally but progressively increases with neurogenesis and reaches its peak in mature, post-migratory neurons [12, 13]. The timing of MeCP2 expression varies by brain region and cell type and correlates closely with the maturation of the given cell type and matches the overall ontogenetic maturation pattern of the CNS [49], i.e., older structures, such as the spinal cord and brain stem, become MeCP2 positive before newer structures, such as the cerebral cortex, hippocampus, and cerebellum [50]. This temporal pattern holds within individual brain regions—e.g., in the cortex, the timing of MeCP2 expression follows the inside-out lamination sequence, with Cajal-Retzius cells becoming positive first, followed by early-born deep-layer neurons, and finally later-born superficial-layer neurons [12]. This suggests that the MeCP2 protein only becomes detectable once the individual neuron reaches a certain point of maturity and is consistent with the hypothesis that MeCP2 plays an important role in maintaining neuronal maturation. Additionally, in the cortex, hippocampus, and the granule cells of the cerebellum, MeCP2 expression is most closely correlated with synaptogenesis, consistent with its proposed role in the formation and maintenance of synapses [50].

In addition to the spatio-temporal dependence of the *MECP2* expression, the brain contains a heterogeneous cell population, including both low MeCP2-expressing cells (MeCP2^lo^) and high MeCP2-expressing cells (MeCP2^hi^), displaying a defined distribution pattern. MeCP2^lo^ cells are present in the highest proportion in the granular layer of the cerebellum, whereas layer IV of the cerebral cortex and the molecular layer of the cerebellum exhibit a higher proportion of MeCP2^hi^ cells. The remaining layers of both regions contain roughly equal proportions of each cell type. The proportion of MeCP2-positive neurons increases throughout postnatal life, and it correlates with the percentage of MeCP2^hi^ cells, indicating a possibly increasing need for MeCP2 function as the nervous system matures [46].

**Fig. 1:**
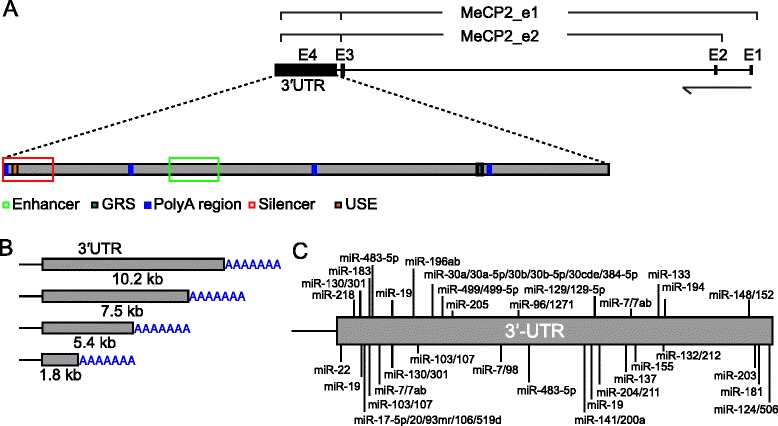
Regulation via the *MECP2* 3'UTR. **a** Schematic depicting the cis-acting regulatory elements in the genomic sequence of the *MECP2* 3'UTR. *Open boxes* indicate the silencer and enhancer described by Liu and Francke [59]. *Closed*, *colored boxes* indicate auxiliary elements involved in polyadenylation, as described by Newnham et al. [58]. *E *exon, *GRS* G-rich element, *USE* upstream sequence element. **b** Four unique *MECP2* transcripts produced by alternative polyadenylation. **c** Relative position of putative miRNA binding sites in the 3'UTR of the 10.2-kb species of *MECP2* mRNA, as defined by Target Scan. This representation is limited to predicted sites for miRNAs that are broadly conserved in vertebrates (with the exception of the human-specific site for miR-483-5p)

Alternative splicing generates two MeCP2 isoforms, MeCP2_e1 and MeCP2_e2, which only differ at their N-termini. Isoform E1 is 498 amino acids long and is translated from exons 1, 3, and 4, whereas isoform E2 is 486 amino acids and translated from exons 2, 3, and 4 [51, 52] (Fig. 1a). These isoforms are also spatio-temporally regulated. The e1 isoform is much more abundant in the brain and also demonstrates more widespread expression throughout development. Early in postnatal life, the e2 transcript is widely expressed but with time becomes largely restricted to the dorsal thalamus and cortical layer V [53]. Consistent with this, a more recent report utilized an MeCP2E1-specific antibody to demonstrate that the E1 protein is also widely expressed, with its highest expression in the cortex and cerebellum, and it is also expressed in high amounts in neurons as compared to astrocytes [54].

It is evident that very intricate regulation is required to achieve the temporal, regional, and cell-population specificity of these expression patterns, and we will now turn our attention to the evidence implicating the 3′UTR as an important contributor to the fine-tuning of homeostatic MeCP2 expression levels throughout development.

### 3′ untranslated region (3′UTR) of *MECP2*

#### Structure and conservation

The *MECP2* gene contains a remarkably large, highly conserved 3′UTR. Coy et al. [55] screened cosmid clones with radioactively labeled cDNA clones they had isolated from human tissue samples and identified a long contig with no open reading frame or introns. They mapped the contig to the 3′UTR of *MECP2*, and further investigation revealed that alternative polyadenylation (poly A) signals could originate transcripts of multiple lengths. Reichwald et al. [56] similarly found by comparative sequence analysis the existence of this additional stretch of sequence 3′ to the previously reported poly(A) site, which terminated in an alternative poly(A) site. In addition, Coy et al. [55] compared the entire human 3′UTR sequence with the mouse sequence and found that homology of this region of the *MECP2* gene (~52 %) is lower than the average for 3′UTRs. However, they discovered blocks of sequence within the 3′UTR that were highly conserved, not only in mouse but also kangaroo, rat, hamster, macaque, and chimpanzee sequences. These regions were also predicted to have high minimum free energy, suggesting a weak secondary structure. Conversely, the regions of the 3′UTR that were not highly similar in primary sequence were predicted to have low minimum free energy, suggesting tight folding. Indeed, the free-energy distribution over the entire sequence was highly similar between human and mouse, suggesting that the overall secondary structure of the 3′UTR was conserved throughout evolution regardless of divergent primary structures between different species and therefore may confer important regulatory functions. Likewise, the loose conformation of the highly conserved regions of sequence suggests that these may represent important binding sites for transregulatory elements. Taken together, such evolutionary analysis emphasizes the importance of the 3′UTR on a very fundamental level.

#### Alternative polyadenylation of MECP2

Alternative poly(A) sites within the 3′UTR can be used to generate four mRNA transcripts of varying length: ~1.8, ~5.4, ~7.5, and ~10.2 kb (Fig. 1b) [57]. Studies have revealed that the distribution and abundance of each transcript varies by tissue and is developmentally regulated. The 10.2-kb transcript is the most abundant in the brain, and in sharp contrast to MeCP2 protein, the expression of this transcript is highly enriched in the embryonic brain but then progressively declines during postnatal development [37, 47], only to be upregulated again in the adult brain [37]. It has been suggested that this decrease in the use of the long 3′UTR transcript accounts for the corresponding increase in MeCP2 protein levels via possible differential regulation conferred by the individual transcripts. However, Samaco et al. [37] found that, while total *MECP2* transcript expression levels across neuronal populations decreased from the fetal to the postnatal stage, the increase in MeCP2 protein expression, along with an increase in the percentage of MeCP2^hi^ cells with age, correlates with an increase in the expression of both the total and the long variant *MECP2* transcripts within the MeCP2^hi^ population. Additionally, they noted a significant decrease in expression of the long *MECP2* transcript within the MeCP2^lo^ population in postnatal brains versus fetal brain and of the ratio of long transcript to total transcripts, which later increased in adult brains. However, the study found no significant correlation between transcript length and MeCP2 protein levels on a single cell level. This highlights how the alternative polyadenylation of the *MECP2* 3′UTR serves as a dynamic source of brain region and even cell-type-specific regulation of MeCP2 expression. While we do not currently fully understand how each individual transcript relates to protein expression, and how this may change with spatio-temporal context, the heterogeneity of the levels of transcript type among cell populations suggests that the 3′UTR may be important for fine-tuning MeCP2 protein expression to meet the homeostatic needs of individual microenvironments in the brain.

Alternative poly(A) is determined both by the presence of pairs of cis-acting core sequences that specify the site of poly(A) as well as auxiliary regulatory elements that can facilitate or repress poly(A) at a designated site. Newnham et al. [58] discovered such cis regulatory elements both upstream and downstream of the binding sites for cleavage and polyadenylation specificity factor (CPSF) in the *MECP2* 3′UTR. They discovered a G-rich element (GRS) downstream of the most proximal poly(A) core sequence (Fig. 1a), the mutation of which resulted in significantly reduced efficiency of polyadenylation. They also showed that this site is specifically bound by hnRNP F, a protein involved in the 3′ end formation, indicating it likely plays an important regulatory role in the production of alternative *MECP2* transcripts. They also found an element upstream of the most distal poly(A) signal, which was very similar to upstream sequence elements (USEs; Fig. 1a) found in human collagen genes and human COX-2. Mutation at this site also reduced polyadenylation efficiency, albeit to a lesser extent. Interestingly, the DNA sequence of the 3′UTR also harbors enhancer and repression elements that act directly on the *MECP2* core promoter (Fig. 1a). These regulatory elements were shown by gel shift assays to bind nuclear proteins, presumably transcription factors [59]. Additional evidence is needed in order to tease out the mechanisms by which these cis elements regulate the transcription and post-transcriptional modification of *MECP2.* Insight into how alternative polyadenylation of *MECP2* is regulated, for example, may shed light on the circumstances under which one transcript is required over another in order to meet the homeostatic needs of the cell.

The complicated nature of the relationship between the length of the *MECP2* 3′UTR and the expression of the MeCP2 protein is likely due to the complex and multifactorial impact of 3′UTR on gene expression. The 3′UTR can play a role in translation efficiency, localization, and the folding and stability of the mRNA [60]. As such, alternative poly(A) offers the ability of the cell to “customize” a transcript to meet its needs. That is, cleaving the transcript at varying poly(A) sites varies the regulatory elements at the level of both the primary and secondary structure, which in turn will affect the stability, localization, translatability, etc. of the mRNA. As such, mutations in the 3′UTR certainly have the potential to affect the stability of *MECP2* transcripts, and indeed, autistic patients carrying non-RTT-causing mutations in conserved sequences of the *MECP2* 3′UTR displayed reduced levels of *MECP2* mRNA compared to controls [61]. Given the fine balance of the MeCP2 expression needed for brain development, this is potentially of high clinical significance.

#### microRNAs (miRNAs) and post-transcriptional regulation of MeCP2

One feature of 3′UTRs that is often of particular interest is the presence of targeting sequences for trans-acting regulatory elements. Among these regulatory elements are miRNAs, which are short, non-coding RNA molecules that are involved in post-transcriptional gene regulation. miRNAs function by base-pairing with a complementary sequence on target messenger RNA (mRNA). This base-pairing is most often imperfect, with exact matching occurring only between nucleotides 2 and 8 (known as the “seed region”) of the miRNA and a complementary sequence in the 3′UTR of the target mRNA [62–64]. This type of partial-binding of miRNA to its target usually results in either translational repression [65], deadenylation [66], or, more rarely, cleavage of the mRNA [67]. miRNAs have also been shown to establish mRNA threshold levels, below which protein translation is highly reduced, thus allowing for a fine-tuning of gene expression levels in addition to overt gene-silencing [68]. miRNAs regulate gene expression in the developing nervous system [69], with roles in regulating neurogenesis [70, 71], neuronal maturation, spinogenesis, dendritic arborization [72], synaptogenesis [73], and neuronal survival [74]. This has profound implications for how miRNA misexpression may affect cognitive capability. For example, Hansen et al. [75] showed that moderate increases in miR-132 in the hippocampus enhanced cognitive capacity, while supra-physiological expression resulted in impaired cognition and an increase in dendritic spines, implying that the decreased capacity for learning and memory resulted from alterations in the structure of synaptic connections.

**Fig. 2:**
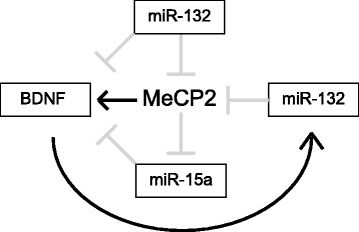
MeCP2 participates in homeostatic feedback loops involving regulation by miRNAs. An example of several feedback mechanisms involving BDNF is depicted here

In line with this, *MECP2* is regulated by several miRNAs (Table 1), and its 3′UTR contains putative target sequences for many more (Fig. 1c); however, the role of miRNAs in regulating *MECP2* expression is diverse and complex. These promiscuous molecules serve as important potential modifiers for local, tissue-, or possibly even cell-type-specific fine-tuning of MeCP2 protein levels, either developmentally or in response to cellular activity. In the nervous system, miRNA-132 has been demonstrated to regulate MeCP2 expression in a homeostatic feedback loop with brain-derived neurotrophic factor (BDNF) [76]. BDNF is a known target of MeCP2, which interacts directly with its promoter to enhance its expression [77]. Klein et al. [76] demonstrated that activation of the cAMP response element-binding protein (CREB) pathway in cortical neurons stimulated an increase in miR-132 expression and subsequent reduction in MeCP2 expression. Blocking miR-132 increased the expression of both MeCP2 and BDNF, while siRNA-mediated knockdown of MeCP2 reduced both BDNF and miR-132 levels, suggesting a potential CREB-mediated regulatory feedback loop (Fig. 2). miR-212, which is closely related to and genetically arranged in tandem with miR-132, and which has been shown to reduce MeCP2 levels in gastric carcinoma cell lines [78], participates in a similar negative feedback loop with MeCP2 in the dorsal striatum [79]. This miR-212-MeCP2 relationship was also shown to have regulatory implications for BDNF, specifically under the conditions of extended cocaine exposure [79]. Chen and colleagues [80] also demonstrated that MeCP2 is a functional target of miR-7b, which is in turn targeted and silenced by MeCP2 as postnatal neurons mature, suggesting another homeostatic regulatory loop. In addition, Han et al. [81] identified a novel, human-specific targeting site for miR-483-5p in the long *MECP2* 3′UTR. They postulated a potential role for this miRNA in fetal development by demonstrating an inverse correlation between elevated miR-483-5p levels and decreased MeCP2 expression levels in human fetal brains. Furthermore, regulation of MeCP2 by miR-124a in the spinal cord may modulate nociception. Kynast et al. [82] demonstrated that peripheral noxious stimulation in mice led to a decrease in miR-124a expression in neurons of the dorsal horn, accompanied by an increase in MeCP2 and pro-inflammatory genes, as well as nociceptive behavior.

**Table 1:** miRNAs that are known to target the *MECP2* 3'UTR and the reported biological significance for each

microRNA	Biological relevance	Reference
miR-132	Homeostatic regulation of MeCP2 and BDNF.	Klein et al. (2007) [76]
	Reduction of miR-132 during ischemic-preconditioning contributes to elevated MeCP2 levels in the cortex following ischemic injury.	Lusardi et al. (2010) [94]
miR-212	Differential regulation of MeCP2 and BDNF in response to prolonged cocaine intake.	Im et al. (2010) [79]
miR-7b	Homeostatic regulation of MeCP2 during postnatal development.	Chen et al. (2014)
miR-483-5p	Human-specific regulation of MeCP2 expression during fetal development.	Han et al. (2013) [81]
miR-124a	May attenuate nociception by repressing MeCP2 and, by extension, downstream pro-inflammatory genes.	Kynast et al. (2013) [82]
miR-22	MeCP2-targeting during ischemic-preconditioning reduces apoptosis and cardiac fibrosis.	Feng et al. (2014) [84]
	Smooth muscle cell differentiation.	Zhao et al. (2015) [83]
miR-511	Only binds *MECP2* 3'UTR carrying C > T SNP. Reduces peripheral MeCP2 expression by ~50 % and has implications for aggression.	Tantra et al. (2014) [93]

The relevance of studying the role of miRNAs in regulating *MECP2* is highlighted further by evidence that miRNAs modulate *MECP2* expression in physiological processes other than neurogenesis. For example, targeting of *MECP2* by miR-22 has been shown to promote smooth muscle cell differentiation [83] and to reduce apoptosis in ischemic cardiomyocytes [84]. Future studies investigating brain-region-specific miRNAs and their potential to target *MECP2* may provide additional insight into the enigmatic relationship between transcript variants and MeCP2 protein expression in different parts of the brain.

### Clinical implications of the *MECP2* 3′UTR beyond RTT

In addition to those causing RTT and related disorders, *MECP2* mutations have been described in other neurodevelopmental disorders, such as autism, and X-linked mental retardation [33, 34, 61, 85, 86]. Some of these mutations occur in the 3′UTR rather than in the coding sequence, in accordance with a need for finely tuned regulation of MeCP2 protein levels for proper neurological function. Coutinho et al. [61] found 21 variations in the *MECP2* 3′UTR of 46 out of 172 autistic patients. Of these variations, 12 did not occur in controls. They also found that *MECP2* mRNA levels in peripheral blood mononuclear cells of four patients with variations in conserved sequences were significantly lower than that of controls, suggesting that changes in at least these conserved sequences may alter mRNA stability and, thus, protein expression levels. Shibayama et al. [86] also found two 3′UTR variations in autistic patients, as well as one variation in a patient with ADHD. While these alterations seem to be most common to autism [61, 86–88], they have also been found in patients with spontaneous intellectual disability [88–90], as well as individuals with atypical RTT, or classic RTT with no detectable pathological mutation in the *MECP2* coding sequence [88, 91]. Mutations in the 3′UTR of *MECP2* were also found in a rare number of patients with RTT in a study by Santos and colleagues [88]; however, none of the five variants they discovered are found in the putative protein-targeting sites of the UTR; additionally, two had been previously described as polymorphisms, one was present in an unaffected father and another was present in an unaffected mother, suggesting that they are not pathogenic, particularly in the case of the father. A summary of these *MECP2* 3′UTR variants and their pathological relevance can be found in Table 2. Taken together, these findings suggest that mutations in the 3′UTR, which would purportedly impact the expression of MeCP2 rather than its function, may not cause full-blown RTT, except in rare cases, but could still impair neurological function given a context allowing for MeCP2 dysregulation.

**Table 2:** Sequence variations in the *MECP2* 3'UTR that have been reported in non-RTT neurological disorders, atypical RTT, or RTT without a detectable pathogenic coding region mutation

Nucleotide change	Disease	Notes	Reference
c.*98insA	1. ADHD		1. Shibayama et al. (2004) [86]
	2. ID		2. Tejada (2006) [29]
	3. PMD delay, ID, autism		3. Santos et al. (2008) [88]
c.*177G > C	Autism		Shibayama et al. (2004) [86]
c.*5348T > C	Autism		Shibayama et al. (2004) [86]
c.*93G > A	ID	Reported in two patients; one also had an intronic variation.	Ylisaukko-oja et al. (2005) [90]
c.*139G > A	Autism with regression		Xi et al. (2007) [87]
c. *371G > C	Autism	Occurs in conserved sequence in patient with reduced *MECP2* mRNA levels.	Coutinho et al. (2007) [61]
c.*554G > A	Autism	Occurs in conserved sequence in patient with reduced *MECP2* mRNA levels.	Coutinho et al. (2007) [61]
c.*2556T > A	Autism	Occurs in conserved sequence in patient with reduced *MECP2* mRNA levels.	Coutinho et al. (2007) [61]
c.*2956G > A	Autism	Occurs in conserved sequence in patient with reduced *MECP2* mRNA levels.	Coutinho et al. (2007) [61]
c.*9G > A	Atypical RTT with ID and autism	No coding *MECP2* mutation.	Santos et al. (2008) [88]
c.*8500C > G; *8503delC	ID, ataxia, epilepsy	C > G variant inherited from unaffected father, deletion from mother.	Santos et al. (2008) [88]
c.473C > T; *14G > A	Atypical RTT	Missense mutation in MBD combined with 3'UTR variation	Santos et al. (2008) [88]
c.*92C > G	RTT	Non-coding *MECP2* mutation	Fendri-Kriaa et al. (2010) [91]

*ID* intellectual disability, *PMD* psychomotor development, *MBD* methyl-CpG-binding domain

In line with this, Hanchard et al. [92] reported an adult male harboring a partial *MECP2* duplication, who was high functioning and able to live independently despite suffering from epilepsy and cognitive impairment. The duplication included all four exons but excluded almost the entire 3′UTR. Given the importance of the 3′UTR for the stability and activity of the MeCP2 protein, the loss of the 3′UTR in the duplicated segment of the *MECP2* gene more likely mitigates the MeCP2 overexpression and, by extension, the severity of symptoms. Additionally, Samaco et al. [37] demonstrated differences in MeCP2 expression levels in autism, pervasive developmental disorder, Prader-Willi syndrome, and Angelman syndrome; by laser-scanning cytometry, the disturbances in MeCP2 expression were determined as due to differential transcriptional and post-transcriptional mechanisms.

miRNA-targeting of the 3′UTR of *MECP2* has also been implicated in neurological dysfunction. Human-specific miR-483-5p, which has been shown to decrease MeCP2 expression, is transcribed from the second intron of the genetically imprinted gene insulin-like growth factor 2 (IGF2). IGF2 expression occurs almost exclusively from the paternal allele, and imprinting defects that lead to expression from the maternal allele cause Beckwith-Wiedemann syndrome (BWS). The study demonstrates that BWS patients with bi-allelic expression of IGF2 also overexpress miR-483-5p and underexpress MeCP2. This finding may have implications for the etiology of higher prevalence of autism in these patients [81]. Additionally, a recent study by Tantra et al. showed that a 50 % overexpression of MeCP2 influences aggression levels in opposing ways in two different strains of mice. To test this interaction between genetic background and expression level in humans, they demonstrated that miR-511, which is expressed in the brain, binds selectively to *MECP2* mRNA transcripts that carry a C > T SNP in the 3′UTR. As a result, T carriers have a ~50 % reduction in peripheral MeCP2 expression. The C allele at this locus is associated with increased aggression in schizophrenia, thus implicating the interaction between altered MeCP2 expression and genetic background as a potential mechanism [93]. Increased expression of MeCP2 resulting from reduction of miR-132-mediated repression has also been implicated in the neuroprotective response to pending ischemic injury [94].

These examples highlight how the 3′UTR may serve as a potential source for pathogenic misregulation of MeCP2, and as such, it may be worthwhile to conduct additional studies investigating the potential pathogenicity of mutations and polymorphisms in this critical region.

## Conclusions

In summary, regulation of MeCP2 expression is complex, multifactorial, and crucial for the proper maintenance and function of the CNS. In this review, we have focused our attention on the role of the *MECP2* 3′UTR in this process. Evidence suggests that the 3′UTR confers multiple levels of regulation on MeCP2 expression that are significant for neurological function. This is consistent with the role of the 3′UTR as seen in other important neural proteins. For example, poly(A) produces two BDNF transcripts, with a long or short 3′UTR. The long 3′UTR represses translation at rest, while the short transcript is actively translated. However, upon neuronal activation, the long transcript, but not the short, undergoes rapid translational activation [95]. Likewise, BDNF is also regulated by miRNAs via its 3′UTR [96].

Post-transcriptional regulation by the 3′UTR offers the advantage of rapid and precise homeostatic control over protein levels in response to a cell’s individual needs. However, the complexity of this single avenue of gene regulation highlights the need for intense study, especially given the fact that a vast array of additional mechanisms exist that manage the expression and function of MeCP2 at every level. For example, the *MECP2* core promoter is located within a CpG island [56], and gene expression levels have been shown to inversely correlate with methylation. In fact, hypermethylation of the *MECP2* core promoter has been observed in autistic patients [97]. Additionally, Liu and Francke [59] identified four enhancers and two silencers within the gene; these enhancers contain predicted binding sites for brain-specific transcription factors, and three of them were able to act in cis with the core promoter. MeCP2 also exists in two isoforms, the alternate expression of which appears to be dependent upon differential methylation of regulatory elements within the *MECP2* promoter, which can be manipulated with decitabine [96]. In addition to tight control over expression, mechanisms such as post-translational modification also contribute to the regulation of MeCP2 activity. For example, activity-dependent phosphorylation of threonine 308 blocks the interaction of MeCP2’s repressor domain with NCoR, which attenuates its transcriptional repression [98]. The advances in understanding the complex and interactive nature of *MECP2* and its regulatory elements are pivotal to unraveling the mechanisms underpinning the development and progression of complex and poorly understood neurodevelopmental disorders and to devise novel therapeutic approaches. miRNAs in particular are attractive therapeutic targets, as there is evidence that their expression can be modified by small molecules [99]. Likewise, methods are being developed to deliver miRNA mimics and/or inhibitors directly into the CNS (e.g., lipid-, polyethylenimine-, and dendrimer-based methods) [100].

Finally, the bulk of the literature exploring the expression, regulation, and function of MeCP2 has focused almost exclusively on neurons, as early studies were not able to show any expression in glial cells. Recently, increasing attention has been pointed at the role played by glial cells in conditioning the morphological and functional development of neurons, and several studies are deciphering the specific roles of glia in neurodevelopment, as well as in RTT and related disorders. However, the relative paucity of information regarding how MeCP2 is regulated in non-neuronal brain cells is a gap that needs to be filled by further investigations.

## Abbreviations

MeCP2: methyl-CpG-binding protein 2
CNS: Central Nervous System
RTT: Rett Syndrome
3'UTR: 3' untranslated region
mRNA: messenger RNA
miRNA: microRNA
poly(A): Polyadenylation
CPSF: Cleavage and polyadenylation specificity factor
GRS: G-rich element
USE: Upstream sequence element
BDNF: Brain derived neurotrophic factor
CREB: cAMP response element-binding protein
IGF2: Insulin-like growth factor 2
BWS: Beckwith-Wiedemann syndrome
